# Four-Rooted Maxillary First Molars: A Systematic Review and Meta-Analysis

**DOI:** 10.1155/2021/8845442

**Published:** 2021-01-20

**Authors:** Gabriel Magnucki, Sven V. K. Mietling

**Affiliations:** ^1^Zahnzentrum Schomaker and Magnucki, Bahnhofstr. 16-18, Bassum 27211, Germany; ^2^Zahnarztpraxis Mietling, Hauptstr. 117, Hille-Oberlübbe 32479, Germany

## Abstract

**Objectives:**

The majority of human maxillary first molars is usually described as having three roots, but different morphologies were documented in several studies and case reports. One very rare and less investigated anatomical anomaly is the occurrence of four radicular structures in the upper first molars. This communication aimed to define the prevalence of four-rooted maxillary first molars on a meta-analytical basis. The external and internal morphology of these teeth was described by the collection of published case reports.

**Materials and Methods:**

Six electronic databases were accessed to collect case reports dealing with four-rooted maxillary first molars, as well as population-based cone-beam computed tomography (CBCT) studies. Afterward, the publications were selected according to predefined inclusion/exclusion criteria and evaluated using the Joanna Briggs Institute Critical Appraisal tool. The teeth of the chosen case reports were then independently analyzed by two dental professionals according to different dental classifications. Furthermore, the population studies were meta-analyzed to calculate the global and regional prevalence of four-rooted maxillary molars.

**Results:**

Included were forty-nine population-based CBCT studies containing 26663 maxillary first molars. Upon these data, the global incidence of four-rooted maxillary molars was meta-analytically determined as 0.047% (95%-CI:0.011–0.103%). In combination with the case reports, it was pointed out that this anomaly is distributed worldwide. Furthermore, forty-eight case reports were included containing fifty-three maxillary molars with four roots. The analyzed teeth exhibited Versiani´s pulpal chamber floor Types A and B. The majority of four-rooted maxillary first molars were classified as Type I regarding Christie's configuration. But, also 7.54% of the altered teeth could not be described by this classification. 62.34% exhibited four root canals, but also variations with five, six, or seven canals were identified. Furthermore, a significant difference was found in the occurrence rate between male and female patients.

**Conclusion:**

Due to the worldwide occurrence, dental professionals should be aware of this rare anomaly to avoid treatment errors, especially during endodontic or surgical therapies.

## 1. Introduction

For centuries, anatomists have investigated dental anatomy to describe each human tooth by determining its coronal and radicular structures. Human maxillary first molars are generally characterized to possess four or five cusps (the additional mesiolingual cusp of Carabelli) and three roots [1, 2]. These roots were designated due to their anatomical position as mesiobuccal, distobuccal, and palatal root, usually exhibiting four root canals (mesiobuccal root with a second canal, whereas the other roots commonly contain only one canal) [3, 4]. However, numerous studies and case reports also presented maxillary first molars with various anatomical alterations resulting in an enormous diversity in the number of roots and canals. For example, rare cases of single, two-, or even five-rooted maxillary first molars were described [3, 5–7] as well as three-rooted versions with multiple canals [8–10].

**Figure 1:**
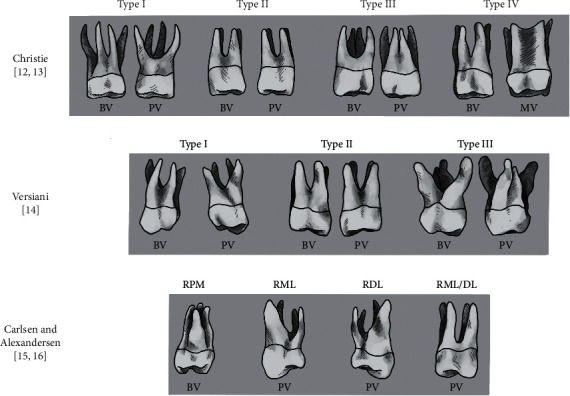
Schematic drawing of radicular classifications of four-rooted maxillary molars modified from [12–16]. Christie classified four-rooted maxillary molars in Type I to Type III based on the radicular shape and the degree of root separation [12]. Type IV was added by Baratto-Filho [13] with a fused mesiobuccal and mesiopalatal root. Versiani's modified radicular classification [14]. Carlsen and Alexandersen defined and named additional radicular structures upon their buccal or palatal location and according to their affinity to the dental crown. BV:buccal view, PV:palatal view, MV:mesial view, RPM:radix paramolaris, RML:radix mesiolingualis, RDL:radix distolingualis, RML/DL:radix mesiolingualis/distolingualis.

Another rare aberration was described by Thews et al. 1979 [11], who identified radiographically four separate roots during endodontic treatment. This unlikely morphological anomaly was classified upon the radicular shape and the degree of root separation by Christie et al. in 1991 [12]. Their characterization was based mainly on studying endodontic treated or extracted maxillary molars and identified three different radicular configurations. Type I maxillary molars have widely divergent, long, and tortuous palatal roots with “cow-horn” shaped buccal roots. Type II maxillary molars have four, shorter, parallel running roots with blunt apices. By definition, “a type III maxillary molar is constricted in root morphology with the mesiobuccal, mesiopalatal, and distopalatal canal encaged in a web of root dentin” [12]. Furthermore, Baratto-Filho et al. in 2002 demonstrated an endodontic case with fused mesiobuccal and mesiopalatal roots and suggested an additional class IV [13] (Figure 1).

However, Versiani et al. in 2012 indicated by studying four-rooted maxillary second molars with micro-CT the Christie's configuration as not feasible, because fusions might occur on a different root level [14]. Therefore, they defined a classification introducing a new type III with less divergent and short palatal roots along with widely divergent buccal roots. In addition, Christie's Types II and III were combined [12, 14]. However, another classification for this very rare anatomical anomaly was designed by naming the additional palatal root as radix mesio- or distolingualis based on its direct affinity to the pronounced part of the crown [15]. Moreover, Carlsen and Alexandersen described maxillary molars exhibiting three buccal roots and characterized the additional radicular structure as radix paramolaris [16] (Figure 1).

Interestingly, all mentioned classifications depended on the investigation of the second or even third maxillary molars and were commonly used for the description of maxillary first molars without any scientific proof [12, 14, 15]. However, due to the rarity of four-rooted maxillary first molars, no anatomical study could be found in dental literature [3]. Therefore, this study systematically collected case reports on maxillary first molars with four roots to study their specific anatomy. In addition, population-based cone-beam computed tomographic (CBCT) studies were analyzed to assess the unknown worldwide prevalence and distribution of these teeth.

## 2. Materials and Methods

### 2.1. Literature Search Strategy

This systematic review, case report collection, and meta-analysis followed the Preferred Reporting Items for Systematic Reviews and Meta-Analyses (PRISMA) [17]. A literature search was conducted between August 2019 and November 2019 by both authors. Six electronic databases were searched (Google Scholar, PubMed, BASE (Bielefeld Academic Search Engine), SciELO (Scientific Electronic Library Online), AJOL (African Journals OnLine), and DHBD (Circumpolar Health Bibliographic Database)) for population-based studies using CBCT imaging for the investigation of maxillary first molars root anatomy according to specific terms (MeSH terms:“maxillary first molar” “cone-beam computed tomography”). Publications in Chinese, English, German, Italian, Japanese, Portuguese, Russian, Spanish, and Turkish were identified. Afterward, the references for these studies were hand-searched. The selection procedure of the records followed a three-step evaluation. At first, titles and abstracts were accessed and characterized as relevant or irrelevant in agreement with predefined inclusion and exclusion criteria (Suppl.  Table 1). Afterward, the full texts (if available) of the selected articles were evaluated according to the mentioned criteria. The resulting articles were critically assessed and evaluated independently by both authors based on the JBI questionnaire [18]. Furthermore, few authors were contacted via e-mail to receive missing information on their studies according to the inclusion criteria and the JBI questions.

**Figure 2:**
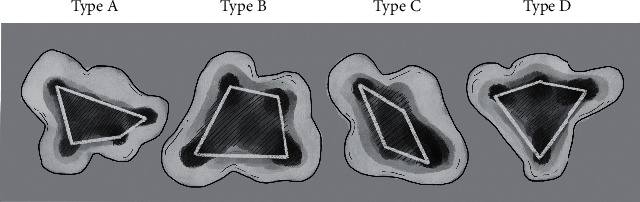
Schematic drawing of Versiani's configuration of canal orifices in four-rooted maxillary second molars [14]. The orifices were classified in relation to the pulpal chamber floor as Type A (irregular quadrilateral-shaped), Type B (trapezoid-shaped), Type C (lozenge-shaped), and Type D (kite-shaped).

Additionally, the mentioned databases were accessed to identify case reports concerning maxillary molars with four roots (MeSH terms:“maxillary first molar” “four roots,” “maxillary first molar” “two palatal roots,” “maxillary first molar” “Radix mesiolingualis,” “maxillary first molar” “Radix distolingualis”). Publications in Chinese, English, Farsi, Portuguese, Spanish, and Turkish were identified. Afterward, the reference lists of the identified case reports were hand-searched. As mentioned above, at first, the titles and abstracts were evaluated according to predefined inclusion and exclusion criteria (Suppl.  Table 1). Second, the available full texts were analyzed upon these criteria. Finally, both authors defined the case report teeth upon Christie's radicular configuration regarding the published radiographs [12]. Afterward, when available, the intraoperative photographs of the teeth were classified according to Versiani's pulpal chamber floor type [14] (Figure 2). Disagreements were resolved through consensus.

### 2.2. Statistical Analysis

The summary of the selected (population-based CBCT) studies and the calculation of the pooled proportion of teeth with 4 roots were carried out by a random-effects model with inverse-variance weights. Since some CBCT studies showed no record of upper first molars with 4 roots, the proportions of individual studies were first transformed for the calculation of the pooled proportion (Freeman-Tukey Double Arcsine Transformation). The graphical representation of the proportions of the individual studies and the fraction pooled share with a 95% confidence interval was carried out with a forest plot. In addition, the dispersion of the studies was illustrated in a funnel plot in which the number of teeth of each study was plotted against the proportion of teeth with 4 root canals. Furthermore, Cohen's kappa was calculated to quantify the degree of compliance of two assessors in answering the JBI questions. All statistical tests were carried out on two sides at the significance level 0.05. Stata/IC 16.1 for Unix (StataCorp 4905 Lakeway Drive, College Station, TX 77845, USA) was used for data preparation and analysis.

## 3. Results

### 3.1. Included Studies

**Figure 3:**
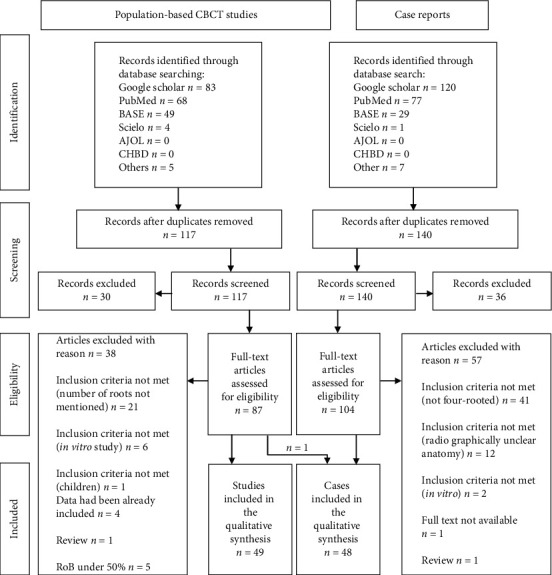
Flowchart summarizing the search strategy and results.

The electronic and manual search identified 117 relevant studies for the population-based CBCT studies. Sixty-eight studies were excluded by accessing the title, abstract, and/or full text (Figure 3). The JBI questions [18] Q5 and Q9 were considered not applicable, and Q6 was eliminated due to the predefined CBCT technique like previous dental studies described [19]. The results of the Cohen kappa interrater reliability for the publications investigated by the JBI questionnaire were 0.883 (Q1), 0.795 (Q2), 0.979 (Q4), 0.645 (Q7), and 0.764 (Q8). The resulting 54 studies had an average score of 77.2%. Five studies were excluded due to their low score (≤50%), whereas 38 papers had a high (≥75%) and 11 a moderate (≥50%) score. The finally selected 49 studies included 26663 investigated maxillary first molars (Table 1).

From 140 selected case reports concerning four-rooted maxillary first molars, 93 publications were excluded based on the predefined inclusion and exclusion criteria. One population-based CBCT study also documented a micro-CT of a four-rooted molar and was included (Figure 3). The finally selected 48 studies included 53 four-rooted maxillary molars (Table 2). In combination with additional information in the CBCT studies, the sample size of the analyzed teeth could be increased. So, 75 maxillary first molars with four roots could be investigated regarding their amount of root canals and 67 teeth to study the gender of the patients. On the other hand, the radicular subtype (46 teeth), Versiani's pulpal chamber floor type (33 teeth), and the distinction of the right or left molar (49 teeth) had a less amount of studied subjects due to missing data in the case reports.

### 3.2. Global Distribution

**Figure 4:**
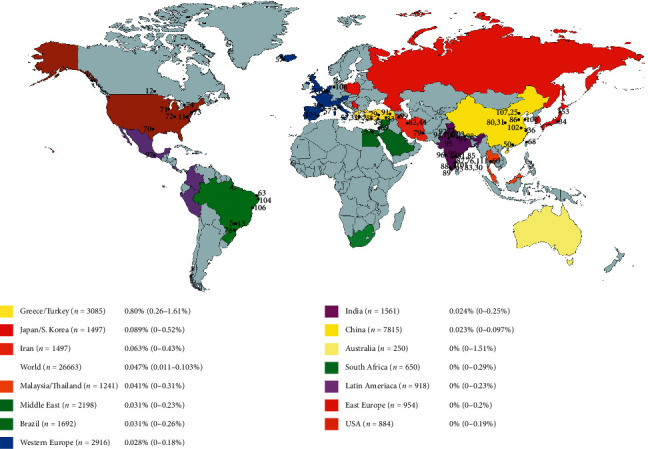
Global distribution of four-rooted maxillary first molars. All case reports and maxillary first molars with four roots identified in CBCT studies were marked on the world map. The regional differences in the occurrence rate were noted.

**Figure 5:**
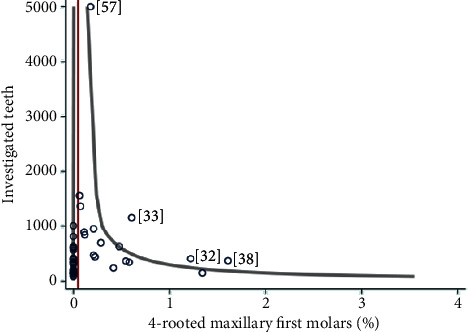
Funnel Plot. The vertical red line marks the median of all studies (0.047). The two gray curves mark the 95% confidence interval, assuming a true percentage of 0.047. The left gray line marks the lower limit of the 95% CI and runs almost parallel to the *y*-axis; i.e., the lower boundary is 0. Overall, the percentage of teeth with 4 roots is small (0.047%); therefore, a large part of the studies shared 0%.

Four-rooted maxillary molars were identified worldwide in population-based CBCT  studies and case reports (Figure 4). The composed prevalence (95% CI) of the analyzed 26663 teeth was calculated as 0.047% (0.011–0.103%) (Table 1). The highest prevalence was meta-analytically found in the Greek-Turkish population with 0.804% (0.255–1.609%). Most of the case reports (43.4%) were documented in India in contrast to the moderate prevalence rate of 0.024% (0–0.249%) (Figure 4, Suppl.  Table 2.1-14). The funnel plot demonstrated that four of the 49 included studies were outside the margins of the 95% confidence interval. This was in the expected proportion of 95% of studies between the curves, resulting in no risk of bias (Figure 5).

### 3.3. Anatomical Description of Four-Rooted Maxillary Molars

The analysis of the case reports (Table 2), according to Christie's radicular structure, demonstrated that 37.7% of the authors used this classification. By using Christie's accurate description for the radiographic identification, we characterized 52.83% as Type I, 18, 87% as Type II, 18, 78% as Type III, 1, and 88% as Type IV, and 7.54% could not be described by this classification (Table 3). Versiani's introduced pulpal chamber floor type was only used by Magnucki et al. in 2018 [61]. Our analysis classified the geometrical location of the root canal orifices in 45.45% as Type A (irregular quadrilateral), 51.52% as Type B (trapezoid), and 3.03% as Type *D* (kite-shaped). Type C could not be found in the investigated case reports (Table 3). The majority of the analyzed teeth (combined CBCT studies and case reports, a sample size of 78 teeth) demonstrated four (62.34%) or five (27.27%) root canals. But also six (9.09%) or even seven (1.30%) root canals were described (Table 3). No statistical difference could be found between the left (50.00%) and right (50.00%) of maxillary first molars with four roots. Of the 68 teeth, where the sex of the patient was mentioned, 60.29% were males, and 39.71% were females. This difference was significant (Table 3). Coronal anomalies or enamel pearls in addition to the morphological alteration of four roots were documented in case reports in 15.1%.

## 4. Discussion

The knowledge of oral anatomy, its anomalies, and their frequencies is fundamental for successful dental therapies. Therefore, this systematic review assessed the prevalence of maxillary first molars with four roots in humans and documented a very low global occurrence rate of 0.047%. Nevertheless, these morphologically altered teeth were found worldwide in documented case reports and population studies, except for Eastern Europe, Australia, and mostly Africa. Probably, a higher study activity in the field of dental anatomy could fill these areas, as it is mainly the lack of publication (e.g., 250 investigated teeth in Australia), which led to these unaddressed geographic regions. Population-based CBCT studies with high amounts of sample size can identify even very rare anatomical anomalies and are therefore a suitable scientific tool [4, 19, 57]. Besides an appropriate number of studied teeth, a reproducible methodology and detailed description of demographic factors are recommended as a guideline for these studies [4, 19]. The present study linked the occurrence of four-rooted maxillary first molars with regionally subgrouped populations based on the demonstrated association of rare morphological variations and ethnicity in dental literature [19, 112]. The highest occurrence rate was found in the Greek-Turkish population with a significantly higher prevalence than in all other populations. The lowest rates were identified in Eastern Europe and on the American continent (Figure 4). However, an anthropological conclusion reconstructing the human prehistoric colonization upon the teeth size as previously shown for C-shaped mandibular second molars [19] could not be demonstrated for four-rooted maxillary first molars.

However, the rarity of four-rooted maxillary first molars underlined by this review (in 26663 investigated maxillary first molars, only 47 exhibited four roots) caused the complete absence of studies concerning this topic [3]. Therefore, a systematic collection of case reports regarding treatment protocols of maxillary molars with four roots was considered to be an appropriate study design. With this technique, the number of 53 teeth could be identified. This amount can be compared to the most extensive published studies on four-rooted maxillary second molars with either 22 [12] or 25 investigated teeth [14]. One of the further strengths of the present review is the combination of CBCT studies and case reports, which results in, e.g., 77 investigated teeth concerning the number of root canals. Thus, upon this data, an anatomical description of four-rooted maxillary molars should be possible.

The radicular structure of four-rooted maxillary first molars is commonly described with Christie's classification (Figure 1), although it was designed mainly upon maxillary second molars [12]. In the present review, 37.7% of the case report`s authors used Christie's characterization. But, even after the application of Christie's defined radiographic regulation, 7.54% of the teeth could not be classified upon the different types. These unclassified teeth had either fusion between roots [94] or three buccal (two mesiobuccal and one distobuccal) *Radixes* [88, 91]. Therefore, Christie's classification cannot be transferred from maxillary second molars with four roots to four-rooted maxillary first molars without neglecting some teeth.

Regarding fusions, this review agreed with Versiani et al. in 2012, who pointed out that Christie's configuration is not feasible because fusions might occur in different levels of all roots (Figure 1) [14]. On the other hand, Versiani's suggested classification also ignored the description of three buccal and one palatal root. But, Versiani investigated four-rooted maxillary second molars, and these teeth exhibit probably no third buccal radicular structure. The main question is whether a second mesiobuccal root could be classified as a mesiopalatal, even if both structures can be found in five-rooted maxillary teeth [113].

However, the definition of Carlsen and Alexandersen, which is rarely used in scientific publications concerning maxillary molars with four roots, included the possibility of three buccal roots but was designed *in vitro* and on untreated teeth [15, 16]. But, their anatomical characterization described the radicular structures based on coronal anomalies [15], which might be decayed under clinical circumstances. In summary, all standard classifications were not entirely feasible or established for the description of four-rooted maxillary first molars. To cover all variants of four- or even five-rooted maxillary molars [113] or O-shaped teeth [114], new classifications have to be designed, which should also support the clinical and radiographic diagnostic.

In relation to Versiani's pulpal chamber floor classification, which defined the localization of the root canal orifices geometrically, the analyzed case reports documented the main allocation on Type A (irregular quadrilateral) and Type B (trapezoid) (Figure 2). With respect to the sample size of 33 teeth, this data corresponds to findings in four-rooted maxillary second molars where Types A and B were exhibited mainly [14]. However, the majority of maxillary first molars with four roots showed one root canal per root, which also correlated with findings in four-rooted maxillary second molars [14]. Five root canals were identified in 27.27% of the cases, but also six or seven canals were found. It must be taken into account that the present review depends mainly on clinical case reports and that *in vivo* CBCT or *in vitro µ*CT studies would have higher reliability.

Interestingly, the present review identified a significant difference in the occurrence of four roots in maxillary first molars between males and females. Comparable data for four-rooted maxillary second molars have not been published due to the rarity of these teeth. Therefore, and under consideration of the sample size of 68 analyzed teeth, the presented data could indicate a morphological discrepancy between the genders, which should be further investigated by worldwide CBCT  population studies as mentioned above. These future anatomical studies should also focus on coronal anomalies (like a pronounced cusp of Carabelli) [15] as well as on the occurrence of enamel pearls in four-rooted maxillary molars. Coronal factors were often mentioned to support the diagnostic of radicular alterations but have to be scientifically proven. In the present review, only 15.1% of the case reports [11, 69, 82, 87, 96, 100, 102, 108] documented further anatomical variations combined with the exhibition of four roots (Table 2). However, dental professionals should be aware of this rare anomaly to avoid treatment errors that result oftentimes in endodontic retreatments [93, 95, 98, 101, 115].

## 5. Conclusion

The occurrence rate for 4-rooted maxillary first molars in humans is 0.047%. By collecting population-based CBCT studies and case reports, this quadrangular anomaly was described with mainly four root canals. The root canal orifices on the pulpal chamber floor are located in trapezoid or irregular quadrilateral shape.

## Figures and Tables

**Table 1:** Analysis of the population-based CBCT studies.

Author	Country	Voxel in *µ*m	CBCT device	*n*	4-Root-ed	In %	CI -95%	Propor. (%)	Additional information to 4-rooted maxillary 1^st^ molars
Zheng et al. 2010 [20]	China	125	Accuitomo (Kyoto, Japan)	627	0	0	0–0.61	2.67	
Zhang et al. 2011 [21]	China	125	Accuitomo	299	0	0	0–1.27	1.43	
Kim et al. 2012 [22]	Korea	167	Dinnova (Gwangmyeong, Korea)	814	0	0	0–0.47	3.26	
Abed et al. 2013 [23]	Iran	150	Planmeca (Helsinki, Finland)	119	0	0	0–3.12	0.61	
Plotino et al. 2013 [24]	Italy	300	NewTom (Verona, Italy)	161	0	0	0–2.33	0.81	
Jing et al. 2014 [25]	China	125	NewTom	630	3	0.476	0.2–1.4	2.68	Two with 4 RCs, 1 with 5 RCs
Silva et al. 2014 [26]	Brazil	200	i-CAT	314	0	0	0–1.21	1.49	
Guo et al. 2014 [27]	USA	150–300	Galileos (Bensheim, Germany)	634	0	0	0–0.6	2.69	
Estrela et a. 2015 [28]	Brazil	100	PreXion (San Mateo, USA)	100	0	0	0–3.7	0.52	
Demırbuğa et al. 2015 [29]	Turkey	75	Newtom	894	1	0.112	0.02–0.6	3.49	Male, 4 RCs
Felsypremila et al. 2015 [30]	India	200	Carestream (Atlanta, USA)	367	2	0.545	0.15–2.0	1.71	Both 4 RCs
Gu et al. 2015 [31]	China	125	Galileos	1365	1	0.073	0.01–0.4	4.65	Male, left maxillary molar, extra root with 1 RC
Nikoloudaki et al. 2015 [32]	Greek	125	NewTom	410	5	1.220	0.52–2.8	1.88	
Altunsoy et al. 2015 [33]	Turkey	300	i-CAT (Hatfield, USA)	1158	7	0.604	0.3–1.2	4.17	4 male, 3 female
Nakazawa et al. 2015 [34]	Japan	300	Aquilion 64 (Otawara, Japan)	443	1	0.226	0.04–1.3	2.01	Female
Beshkenadze and Chipashvili 2015 [35]	Georgia	85–133	Gendex (DesPlaines,USA)	221	0	0	0–1.71	1.09	
Tian et al. 2016 [36]	China	160	NewTom	1558	1	0.064	0.011–0.363	5.04	4 RCs
Tanvi et al. 2016 [37]	India	76	i-CAT	201	0	0	0–1.875	1.00	
Kalender et al. 2016 [38]	Cyprus	170	NewTom	373	6	1.609	0.74–3.5	1.74	5 male, 1 female, extra root in 80% 4 RC, in 20% 5 RCs
Monsarrat et al. 2016 [39]	France	200	Carestream	149	2	1.342	0.369–4.762	0.76	Both 5 RCs, 1 left and 1 right maxillary molar
Naseri et al. 2016 [40]	Iran	200	NewTom	149	0	0	0–2.513	0.76	
Liu and Ma 2016 [41]	China	200	Carestream	83	0	0	0–4.424	0.43	
Irhaim 2016 [42]	South A.	150	Galileos	400	0	0	0–0.951	1.84	
Mohan et al. 2017 [43]	India	100–200	Planmeca	143	0	0	0–2.616	0.73	
Ghoncheh et al. 2017 [44]	Iran	300	NewTom	345	2	0.580	0.16–2.1	1.62	Both 4 RCs
Khademi et al. 2017 [45]	Iran	150	Galileos	389	0	0	0–0.978	1.80	
Lin et al. 2017 [46]	Taiwan	250	i-CAT	196	0	0	0–1.922	0.98	
Olczak and Pawlicka 2017 [47]	Poland	125	Gendex	185	0	0	0–2.034	0.93	
Pérez-Heredia et al. 2017 [48]	Spain	180	Carestream	142	0	0	0–2.634	0.72	
Ghobashy et al. 2017 [49]	Egypt	133	Cranex (Tuusula, Finland)	605	0	0	0–0.631	2.59	
Wang et al. 2017 [50]	China	200	Planmeca	953	2	0.210	0.06–0.8	3.65	1 with 4 RCs, 2 left molars
Al-Shehri et al. 2017 [51]	Saudi-A.	300	Different	351	0	0	0–1.083	1.65	
Al-Kadhim et al. 2017 [52]	Malaysia	300–600	Accuitomo	421	0	0	0–0.90	1.92	
Ogawa and Seki 2017 [53]	Japan	160	Planmeca	240	1	0.417	0.07–2.3	1.17	
Zhang et al. 2017 [54]	China	150	NewTom	1008	0	0	0–0.38	3.80	
Martins et al. 2018 [55]	Portugal	200	Planmeca	567	0	0	0–0.67	2.46	
Razumova et al. 2018 [56]	Russia	300	3D eXam (Hatfield,USA)	410	0	0	0–0.93	1.88	
Martins et al. 2018 [57]	Worldw.	75–250	Different	5000	9	0.180	0.10–0.3	8.59	2 with 4 RCs, 7 with 5 RCs
Salem et al. 2018 [58]	Egypt	150	Planmeca	138	0	0	0–2.71	0.70	
Arbildo Villalta 2018 [59]	Peru	125	Planmeca	168	0	0	0–2.235	0.85	
Ratanajirasut et al. 2018 [60]	Thailand	100	Accuitomo	476	1	0.210	0.04–1.2	2.13	4 RCs
Salzmann 2018 [61]	Austria	80	Accuitomo	147	0	0	0–2.55	0.75	
Pan et al. 2019 [62]	Malysia	250	3D eXam	344	0	0	0–1.10	1.62	
Candeiro et al. 2019 [63]	Brazil	125	Prexion	700	2	0.286	0.08–1.0	2.90	
Mohara et al. 2019 [64]	Brazil	125	Accuitomo	328	0	0	0–1.16	1.55	
Kewalramani et al. 2019 [65]	India	180	Carestream	600	0	0	0–0.64	2.57	
Popović et al. 2019 [66]	Serbia	160	Galileos	138	0	0	0–2.71	0.70	
Mashyakhy and gambarini 2019 [67]	Saudi-A.	250	Accuitomo	354	0	0	0–1.07	1.66	
Tzeng et al. 2019 [68]	Taiwan	250	i-CAT	846	1	0.118	0.02–0.7	3.35	Male, 5 RCs, left
World	Overall random pooled*∗*			**26663**	**47**	**0.047**	**0.011–0.103**	**100**	

CBCT:cone-beam computed tomographic, Propor.:proportion, RC:root canal, Saudi-A.:Saudi Arabia, South A.:South Africa, Worldw.:worldwide.

**Table 2:** Analysis of the case reports.

Author	Country	Age	Sex	Tooth number	Root canals				Christies's radicular type [12, 13]	Versiani's pulpal floor type [14]	Additional information
					*MB*	*DB*	*MP*	*DP*			
Thews et al. 1979 [11]	USA	21	M	n.m.	1	1	1	1	n.m. (I)	—	Single, enlarged lingual cusp
Stabholz and Friedman 1983 [69]	Israel	13	F	16	2	1	1	1	n.m. (III)	B	Unusual configuration of the crown
Wong et al. 1991 [70]	USA	22	M	26	2	1	2	1	n.m. (III)	—	
Christie et al. 1991 [12]	Canada	n.m.	F	16	1	1	1	1	I	B	
Jacobsen and Nii 1994 [71]	USA	25	M	26	2	1	1	1	n.m. (III)		Presented case 3
Di Fiore 1999 [72]	USA	31	M	16	1	1	1	1	II	B	
Baratto-Filho et al. 2002 [13]	Japan	38	F	16	1	1	1	1	I	—	Presented case 1
Maggiore et al. 2002 [73]	USA	19	M	26	2	1	2	1	n.m. (III)	—	Third palatal canal
Barbizam et al. 2004 [5]	Brazil	35	M	26	1	1	1	1	I	—	Presented case 1
Nahmias and Bery 2006 [74]	Canada	58	F	26	1	1	1	1	n.m. (I)	—	
Adanir 2007 [75]	Turkey	31	M	16	2	2	1	1	n.m. (II)	A	
Ravishanker and Prashanthi 2008 [76]	India	25	F	26	1	1	1	1	n.m. (I)	—	
Gandhi and Madan 2009 [77]	India	50	M	26	1	1	1	1	n.m. (II)	B	
Tomazinho et al. 2010 [78]	Brazil	32	M	26	2	2	1	1	I	B	MB and DB RC : Vertucci Class II
Salapoor and Mollashahi 2010 [79]	Iran	40	F	n.m.	1	1	1	1	n.m. (II)	—	
He et al. 2010 [80]	China	35	M	16	1	1	1	1	n.m. (II)	A	
Chakradhar Raju et al. 2010 [81]	India	24	M	26	1	1	1	1	I	B	
	India	21	M	16	1	1	1	1	I	—	
Moghaddas and Tabari 2010 [82]	Iran	41	F	26	1	1	1	1	n.m. (I)	—	Enamel pearl, hemisection
Kottoor et al. 2011 [83]	India	42	M	16	1	1	1	1	n.m. (III)	A	2 fused palatal roots
Singh et al. 2011 [84]	India	21	F	16	1	1	1	1	n.m. (I)	A	Presented case 1
Reddy et al. 2011 [85]	India	45	M	16	1	1	1	1	n.m. (I)	B	
Du et al. 2011 [86]	China	21	F	26	2	1	1	1	n.m. (III)	B	
Madhuram et al. 2012 [87]	India	27	F	n.m.	1	1	1	1	n.m. (III)	—	Pronounced Carabelli tubercle
Kottoor et al. 2012 [88]	India	23	M	26	1	1	1	1	n.d.	A	second mesiobuccal root, 16 with identical morphology
	India	23	M	16	n.m	n.m.	n.m.	n.m.	n.d.	—	
Mathew et al. 2013 [89]	India	35	M	16	1	1	1	1	I	—	
Rajalbandi et al. 2013 [90]	India	42	M	26	1	1	1	1	I	B	
Yesidal Yeter et al. 2013 [91]	Turkey	28	M	16	2	2	1	1	n.d.	A	second mesiobuccal root
Aggarwal et al. 2013 [92]	India	24	M	26	1	1	1	1	I	A	
Ghani et al. 2013 [93]	India	34	F	16	1	1	1	1	I	B	End. retreatment, both teeth fused MB&MP roots
	India	34	F	26	1	1	1	1	I	B	
Sharma et al. 2014 [94]	India	31	F	16	1	2	1	1	n.d.	D	Fused MB&MP and DB&DP roots
Kararia et al. 2014 [95]	India	20	F	26	1	2	1	1	I	B	Endodontic retreatment, DB canal:Vertucci Class II
Shah and Jadhav 2014 [96]	India	38	F	16	1	1	1	1	n.m. (II)	B	Crown with four palatal cusps
Sánchez-Salas et al. 2014 [97]	Mexico	31	M	26	1	1	1	1	I	B	
Shrestha 2015 [98]	Nepal	58	F	26	2	1	1	1	II	A	Presented case 1, End. Retreatment
Asghari et al. 2015 [99]	Iran	21	F	16	1	1	1	1	I	B	
Wu and Wu 2015 [100]	China	29	M	26	1	1	1	1	n.m. (II)	B	
	China	37	M	16	1	1	1	1	n.m. (I)	v	Enamel pearl, pronounced cusp of Carabelli
Gu et al. 2015 [31]	China	n.a.	n.a.	16	1	2	1	2	n.m. (IV)	—	Fused MB&MP roots
Deepa et al. 2016 [101]	India	41	F	16	1	1	1	1	n.m. (I)	—	CBCT after extraction
	India	n.m.	n.m.	n.m.	1	1	1	1	I	A	End. Retreatment, DB canal: Vertucci II
Tao et al. 2016 [102]	China	10	F	16	1	1	1	1	n.m. (II)	A	Three well-developed lobulated palatal cusps
Nayak et al. 2016 [103]	India	24	M	16	1	1	1	1	I	A	
Barreto and Lins 2016 [104]	Brazil	28	M	26	1	1	1	1	n.m. (I)	—	MB canal: Vertucci Type II
Vázquez and Llácer 2016 [105]	Spain	46	M	16	2	1	1	1	n.m. (I)	A	
Rodrigues et al. 2016 [106]	Brazil	23	F	16	3	2	1	1	n.m. (III)	A	
Cao et al. 2017 [107]	China	30	M	16	2	1	1	1	n.m. (II)	B	
Magnucki et al. 2018 [108]	Germany	51	M	26	2	1	1	1	I	A	2 enamel pearls, MB RC : Vertucci II
Meena and Hasija 2018 [109]	India	27	F	16	1	1	1	1	n.m. (III)	—	
Schryvers et al. 2018 [110]	Belgium	44	M	26	1	1	1	1	I	A	
Sriganesh and Priyan 2019 [111]	India	18	F	26	2	1	1	1	n.m. (III)	—	

DB :distobuccal, DP:distopalatal, End.:endodontic, F:female, *M:* male, MB:mesiobuccal, MP:mesiopalatal, n.d.:not defined, n.m. :not mentioned, RC:root canal.

**Table 3:** Evaluation of different anatomical characteristics, classification, and gender.

Christie's Radicular Type (*n* = 46) [12, 13]	Type I	52.83%
	Type II	18.87%
	Type III	18.87%
	Type IV	1.88%
	n.d.	7.54%
Pulpal floor type (*n* = 33) [14]	Type A	45.45%
	Type B	51.52%
	Type C	0.00%
	Type D	3.03%
Amount of root canals (*n* = 77)	4 RCs	62.34%
	5 RCs	27.27%
	6 RCs	9.09%
	7 RCs	1.30%
Tooth number (*n* = 54)	Left #26	50.0%
	Right #16	50.0%
Sex (*n* = 68)	Female	39.71%
	Male	60.29%*∗*

The information on the investigated case reports and the CBCT studies was combined. *∗p* < 0.05, n.d.:not defined, RC:root canal.

## Data Availability

All underlying data can be found in the manuscript.

## Supplementary Materials

Supplementary Materials Supplementary Table 1:the predefined inclusion and exclusion criteria for the literature selection of population-based CBCT  studies and case reports concerning four-rooted maxillary first molars. Supplementary Table 2: the investigated CBCT studies in regional subgroups to evaluate the worldwide occurrence rate of maxillary first molars with four roots. The final results were illustrated in Figure 4. The analyzed regional subgroups are in alphabetical order Australia (Suppl. Table 2.10), Brazil (Suppl. Table 2.6), China (Suppl. Table 2.9), East Europe (Suppl. Table 2.13), Greece/Turkey/Cyprus (Suppl. Table 2.1), India (Suppl. Table 2.8), Iran (Suppl. Table 2.3), Japan/South Korea (Suppl. Table 2.2), Latin America (Suppl. Table 2.12), Malaysia/Thailand (Suppl. Table 2.4), Middle East (Suppl. Table 2.5), Western Europe (Suppl. Table 2.7), South Africa (Suppl. Table 2.11), and USA (Suppl. Table 2.14).
