# Supplementary material for: Four-Rooted Maxillary First Molars: A Systematic Review and Meta-Analysis
Source: Int J Dent. 2021 Jan 20;2021:8845442. doi: 10.1155/2021/8845442 (PMC7843171; doi:10.1155/2021/8845442)
Supplement: Supplementary Materials — Supplementary Table 1:the predefined inclusion and exclusion criteria for the literature selection of population-based CBCT studies and case reports concerning four-rooted maxillary first molars. Supplementary Table 2: the investigated CBCT studies in regional subgroups to evaluate the worldwide occurrence rate of maxillary first molars with four roots. The final results were illustrated in Figure 4. The analyzed regional subgroups are in alphabetical order Australia (Suppl. Table 2.10), Brazil (Suppl. Table 2.6), China (Suppl. Table 2.9), East Europe (Suppl. Table 2.13), Greece/Turkey/Cyprus (Suppl. Table 2.1), India (Suppl. Table 2.8), Iran (Suppl. Table 2.3), Japan/South Korea (Suppl. Table 2.2), Latin America (Suppl. Table 2.12), Malaysia/Thailand (Suppl. Table 2.4), Middle East (Suppl. Table 2.5), Western Europe (Suppl. Table 2.7), South Africa (Suppl. Table 2.11), and USA (Suppl. Table 2.14). [file 8845442.f1.docx]

**Supplementary Material**

|  | **Population based CBCT studies** | **Case Reports** |
| --- | --- | --- |
| **Inclusion criteria** | Evaluation with CBCT  *in vivo* study  Human Study in adults  Maxillary first molars investigated  Sample size of teeth documented  Number of roots mentioned  Population or country of the study are given | Human Case report  Endodontic treatment  Periodontal treatment  Oral surgical procedure  CBCT scan  Maxillary first molars with four roots  Clear documentation of four roots (standard radiograph or CBCT)  Country of the study are given |
| **Exclusion criteria** | Review studies  Data has been already included in other study  Deciduous teeth  *in vitro* study  Full text not available | Review studies  Data has been already included in other study  *in vitro* study  Maxillary molars with less or more than four roots  Radiographically unclear dental anatomy  Full text not available |

**Suppl. Table 1:** Pre-defined inclusion and exclusion criteria.

**Table 2.1: Turkey/Greece/Cyprus**

| Study | Country | Teeth | 4-root | (%) | CI-95% | (%) |
| --- | --- | --- | --- | --- | --- | --- |
| Demirbuga et al. 2014 [29] | Turkey | 894 | 1 | 0.112 | 0.020-0.631 | 23.2 |
| Nikoloudaki et al. 2015 [32] | Greece | 410 | 5 | 1.220 | 0.522-2.823 | 18.9 |
| Altunsoy et al. 2015 [33] | Turkey | 1158 | 7 | 0.604 | 0.293-1.242 | 24.3 |
| Kalender et al. 2016 [38] | Cyprus | 373 | 6 | 1.609 | 0.739-3.464 | 18.2 |
| Martins et al. 2018 [57] | Greece | 250 | 4 | 1.600 | 0.624-4.041 | 15.5 |
| Overall (random effects) | Turkey/Greece | 3085 | 23 | 0.804 | 0.255-1.609 | 100 |

**Table 2.2: Japan/South Korea**

| Study | Country | Teeth | 4-root | (%) | CI-95% | (%) |
| --- | --- | --- | --- | --- | --- | --- |
| Kim et al. 2012 [22] | Korea | 814 | 0 | 0 | 0-0.470 | 44.5 |
| Nakazawa et al. 2015 [34] | Japan | 443 | 1 | 0.226 | 0.040-1.267 | 33.1 |
| Ogawa & Seki 2017 [39] | Japan | 240 | 1 | 0.417 | 0.074-2.322 | 22.4 |
| Overall (random effects) | Japan | 1497 | 2 | 0.089 | 0-0.517 | 100 |

**Table 2.3: Iran**

| Study | Country | Teeth | 4-root | (%) | CI-95% | (%) |
| --- | --- | --- | --- | --- | --- | --- |
| Abed et al. 2013 [23] | Iran | 119 | 0 | 0 | 0-3.127 | 11.9 |
| Naseri et al. 2016 [40] | Iran | 149 | 0 | 0 | 0-2.513 | 14.9 |
| Ghoncheh et al. 2017 [44] | Iran | 345 | 2 | 0.580 | 0.159-2.089 | 34.4 |
| Khademi et al. 2017 [45] | Iran | 389 | 0 | 0 | 0-0.978 | 38.8 |
| Overall (random effects) | Iran | 1002 | 2 | 0.063 | 0-0.432 | 100 |

**Table 2.4: Malaysia/Thailand**

| Study | Country | Teeth | 4-root | (%) | CI-95% | (%) |
| --- | --- | --- | --- | --- | --- | --- |
| Al-Kadhim et al. 2017 [52] | Malaysia | 421 | 0 | 0 | 0-0.904 | 33.9 |
| Ratanajirasut et al. 2018 [56] | Thailand | 476 | 1 | 0.210 | 0.037-1.180 | 38.4 |
| Pan et al. 2019 [62] | Malaysia | 344 | 0 | 0 | 0-1.104 | 27.7 |
| Overall (random effects) | Malaysia/Thailand | 1241 | 1 | 0.041 | 0-0.305 | 100 |

**Table 2.5: Middle East**

| Study | Country | Teeth | 4-root | (%) | CI-95% | (%) |
| --- | --- | --- | --- | --- | --- | --- |
| Ghobashy et al. 2017 [49] | Egypt | 605 | 0 | 0 | 0-0.631 | 26.3 |
| Al-Shehri et al. 2017 [51] | Saudi-Arabia | 351 | 0 | 0 | 0-1.083 | 16.0 |
| Martins et al. 2018 [57] | Egypt | 250 | 1 | 0.400 | 0.071-2.231 | 11.6 |
| Martins et al. 2018 [57] | Kuwait | 250 | 2 | 0.800 | 0.220-2.869 | 11.6 |
| Martins et al. 2018 [57] | Syria | 250 | 0 | 0 | 0-1.513 | 11.6 |
| Salem et al. 2018 [58] | Egypt | 138 | 0 | 0 | 0-2.708 | 6.6 |
| Mashyakhy & Gambarini 2019 [67] | Saudi-Arabia | 354 | 0 | 0 | 0-1.074 | 16.1 |
| Overall (random effects) | Middle East | 2198 | 3 | 0.031 | 0-0.225 | 100 |

**Table 2.6: Brazil**

| Study | Country | Teeth | 4-root | (%) | CI-95% | (%) |
| --- | --- | --- | --- | --- | --- | --- |
| Silva et al. 2014 [26] | Brazil | 314 | 0 | 0 | 0-1.209 | 18.6 |
| Estrela et a. 2015 [28] | Brazil | 100 | 0 | 0 | 0-3.699 | 5.9 |
| Martins et al. 2018 [57] | Brazil | 250 | 0 | 0 | 0-1.513 | 14.8 |
| Candeiro et al. 2019 [63] | Brazil | 700 | 2 | 0.286 | 0.078-1.036 | 41.3 |
| Mohara et al. 2019 [64] | Brazil | 328 | 0 | 0 | 0-1.158 | 19.4 |
| Overall (random effects) | Brazil | 1692 | 2 | 0.031 | 0-0.254 | 100 |

**Table 2.7: Western Europe**

| Study | Country | Teeth | 4-root | (%) | CI-95% | (%) |
| --- | --- | --- | --- | --- | --- | --- |
| Plotino et al. 2013 [24] | Italy | 161 | 0 | 0 | 0-2.330 | 5.5 |
| Monsarrat et al. 2016 [39] | France | 149 | 2 | 1.342 | 0.369-4.762 | 5.1 |
| Perez-Heredia et al. 2017 [48] | Spain | 142 | 0 | 0 | 0-2.634 | 4.9 |
| Martins et al. 2018 [55] | Portugal | 567 | 0 | 0 | 0-0.673 | 19.4 |
| Martins et al. 2018 [57] | Belgium | 250 | 0 | 0 | 0-1.513 | 8.6 |
| Martins et al. 2018 [57] | Netherland | 250 | 0 | 0 | 0-1.513 | 8.6 |
| Martins et al. 2018 [57] | Spain | 250 | 1 | 0.400 | 0.071-2.231 | 8.6 |
| Martins et al. 2018 [57] | Italy | 250 | 0 | 0 | 0-1.513 | 8.6 |
| Martins et al. 2018 [57] | Iceland | 250 | 1 | 0.400 | 0.071-2.231 | 8.6 |
| Martins et al. 2018 [57] | France | 250 | 0 | 0 | 0-1.513 | 8.6 |
| Martins et al. 2018 [57] | England | 250 | 0 | 0 | 0-1.513 | 8.6 |
| Salzmann 2018 [61] | Austria | 147 | 0 | 0 | 0-2.547 | 5.0 |
| Overall (random effects) | Western Europe | 2916 | 4 | 0.028 | 0-0.183 | 100 |

**Table 2.8: India**

| Study | Country | Teeth | 4-root | (%) | CI-95% | (%) |
| --- | --- | --- | --- | --- | --- | --- |
| Felsypremila et al. 2015 [30] | India | 367 | 2 | 0.545 | 0.150-1.965 | 23.5 |
| Tanvi et al. 2016 [36] | India | 201 | 0 | 0 | 0-1.875 | 12.9 |
| Mohan et al. 2017 [43] | India | 143 | 0 | 0 | 0-2.616 | 9.2 |
| Martins et al. 2018 [57] | India | 250 | 0 | 0 | 0-1.513 | 16.0 |
| Kewalramani et al. 2019 [65] | India | 600 | 0 | 0 | 0-0.636 | 38.4 |
| Overall (random effects) | India | 1561 | 2 | 0.024 | 0-0.249 | 100 |

**Table 2.9: China**

| Study | Country | Teeth | 4-root | (%) | CI-95% | (%) |
| --- | --- | --- | --- | --- | --- | --- |
| Zheng et al. 2010 [20] | China | 627 | 0 | 0 | 0-0.609 | 8.0 |
| Zhang et al. 2011 [21] | China | 299 | 0 | 0 | 0-1.268 | 3.8 |
| Jing et al. 2014 [25] | China | 630 | 3 | 0.476 | 0.162-1.391 | 8.1 |
| Gu et al. 2015 [31] | China | 1365 | 1 | 0.073 | 0.013-0.414 | 17.5 |
| Tian et al. 2016 [36] | China | 1558 | 1 | 0.064 | 0.011-0.363 | 19.9 |
| Liu & Ma 2016 [41] | China | 83 | 0 | 0 | 0-4.424 | 1.1 |
| Lin et al. 2017 [46] | Taiwan | 196 | 0 | 0 | 0-1.922 | 2.5 |
| Wang et al. 2017 [50] | China | 953 | 2 | 0.210 | 0.058-0.762 | 12.2 |
| Zhang et al. 2017 [54] | China | 1008 | 0 | 0 | 0-0.380 | 12.9 |
| Martins et al. 2018 [57] | China | 250 | 0 | 0 | 0-1.513 | 3.2 |
| Tzeng et al. 2019 [68] | Taiwan | 846 | 1 | 0.118 | 0.021-0.666 | 10.8 |
| Overall (random effects) | China | 7815 | 8 | 0.023 | 0-0.097 | 100 |

**Table 2.10: Australia**

| Study | Country | Teeth | 4-root | (%) | CI-95% | (%) |
| --- | --- | --- | --- | --- | --- | --- |
| Martins et al. 2018 [57] | Australia | 250 | 0 | 0 | 0-1.513 |  |

**Table 2.11: South Africa**

| Study | Country | Teeth | 4-root | (%) | CI-95% | (%) |
| --- | --- | --- | --- | --- | --- | --- |
| Irhaim 2016 [42] | South Africa | 400 | 0 | 0 | 0-0.951 | 61.5 |
| Martins et al. 2018 [57] | South Africa | 250 | 0 | 0 | 0-1.513 | 38.5 |
| Overall (random effects) | South Africa | 650 | 0 | 0 | 0-0.288 | 100 |

**Table 2.12: Latin America**

| Study | Country | Teeth | 4-root | (%) | CI-95% | (%) |
| --- | --- | --- | --- | --- | --- | --- |
| Martins et al. 2018 [57] | Mexiko | 250 | 0 | 0 | 0-1.513 | 27.2 |
| Martins et al. 2018 [57] | Venezuela | 250 | 0 | 0 | 0-1.513 | 27.2 |
| Martins et al. 2018 [57] | Costa Rica | 250 | 0 | 0 | 0-1.513 | 27.2 |
| Arbildo Villalta 2018 [59] | Peru | 168 | 0 | 0 | 0-2.235 | 18.3 |
| Overall (random effects) | Latin America | 918 | 0 | 0 | 0-0.231 | 100 |

**Table 2.13: East Europe**

| Study | Country | Teeth | 4-root | (%) | CI-95% | (%) |
| --- | --- | --- | --- | --- | --- | --- |
| Beshkenadze & Chipashvili 2015 [35] | Georgia | 221 | 0 | 0 | 0-1.709 | 23.2 |
| Olczak & Pawlicka 2017 [47] | Poland | 185 | 0 | 0 | 0-2.034 | 19.4 |
| Razumova et al. 2018 [56] | Russia | 410 | 0 | 0 | 0-0.928 | 42.9 |
| Popovic et al. 2019 [66] | Serbia | 138 | 0 | 0 | 0-2.708 | 14.5 |
| Overall (random effects) | East Europe | 954 | 0 | 0 | 0-0.195 | 100 |

**Table 2.14: USA**

| Study | Country | Teeth | 4-root | (%) | CI-95% | (%) |
| --- | --- | --- | --- | --- | --- | --- |
| Guo et al. 2014 [27] | USA | 634 | 0 | 0 | 0-0.602 | 71.7 |
| Martins et al. 2018 [57] | USA | 250 | 0 | 0 | 0-1.513 | 28.3 |
| Overall (random effects) | USA | 884 | 0 | 0 | 0-0.191 | 100 |

**Suppl. Table 2.1-14:** Regional sub-grouped evaluation of the occurrence rate of maxillary first molars with four roots.
